# Angry, old, male – and trustworthy? How expressive and person voice characteristics shape listener trust

**DOI:** 10.1371/journal.pone.0232431

**Published:** 2020-05-04

**Authors:** Annett Schirmer, Man Hey Chiu, Clive Lo, Yen-Ju Feng, Trevor B. Penney

**Affiliations:** 1 Department of Psychology, The Chinese University of Hong Kong, Shatin, Hong Kong; 2 The Brain and Mind Institute, The Chinese University of Hong Kong, Shatin, Hong Kong; 3 Department of Psychology, National Taiwan University, Taipei, Taiwan; Arizona State University, UNITED STATES

## Abstract

This study examined how trustworthiness impressions depend on vocal expressive and person characteristics and how their dependence may be explained by acoustical profiles. Sentences spoken in a range of emotional and conversational expressions by 20 speakers differing in age and sex were presented to 80 age and sex matched listeners who rated speaker trustworthiness. Positive speaker valence but not arousal consistently predicted greater perceived trustworthiness. Additionally, voices from younger as compared with older and female as compared with male speakers were judged more trustworthy. Acoustic analysis highlighted several parameters as relevant for being perceived as trustworthy (i.e., accelerated tempo, low harmonic-to-noise ratio, more shimmer, low fundamental frequency, more jitter, large intensity range) and showed that effects partially overlapped with those for perceived speaker affect, age, but not sex. Specifically, a fast speech rate and a lower harmonic-to-noise ratio differentiated trustworthy from untrustworthy, positive from negative, and younger from older voices. Male and female voices differed in other ways. Together, these results show that a speaker’s expressive as well as person characteristics shape trustworthiness impressions and that their effect likely results from a combination of low-level perceptual and higher-order conceptual processes.

## Introduction

Trust is essential for cooperation to emerge both at a small scale, such as among family members and friends, and at a larger scale, such as among groups within a society. Thus, recognizing trustworthy partners who serve not only themselves but are likely to engage in reciprocity is of individual and communal benefit. Although it is well known that humans aim to maximize this benefit by gauging the looks and behaviors of others [1,2], the underlying mechanisms are still poorly understood. Moreover, it remains unclear whether and how vocal signals are relevant. Here, we sought to better understand their role and to explore what aspects of a speaker’s voice bias listener trust. Specifically, we investigated how vocal affect and person characteristics (i.e., age, sex) shape trustworthiness perceptions and examined associated acoustical patterns.

The vocal apparatus, that is the instrument from which the human voice emanates, is a complex system comprising a wind generator—the lungs, a string-like vibrator—the vocal folds, and a sequence of resonance chambers such as the pharynx, mouth and nasal cavities [3]. Relatively fixed structural aspects (e.g., vocal tract length, vocal fold size) as well as dynamic bodily activity (e.g., breathing, muscle tension) modulate the workings of the vocal apparatus and thus how the voice sounds. Resulting inter- and intra-individual differences can be quantified by a range of acoustic measures, also referred to as acoustic parameters, that describe a sound’s frequency, intensity, and duration characteristics.

A substantial body of research has demonstrated statistical relationships between certain acoustic profiles and the affective state that listeners attribute to a speaker [4–6]. For example, fundamental frequency (F0), which is perceived as pitch or voice melody, is higher for elation as compared with other emotions such as anger and sadness [4]. Additionally, there is evidence that voice acoustics predict listener impressions of more stable person characteristics [7–14] including the extent to which a speaker appears trustworthy. Specifically, a lower mean F0 in speech has been repeatedly linked to increased perceived kindness or trustworthiness [8,15–18]. Additionally, it has been shown that utterances rated as high and low on trustworthiness differ in F0 contour [19]. For example, in one study highly trustworthy pronunciations of the word “hello” were characterized by a rising F0 from the first to the second syllable. Moreover, when applied to other words, this rising F0 contour was found to influence listeners in choosing the more trustworthy of two voices [20].

However, not all research corroborates these observations and attests to a simple relationship between trustworthiness and F0. For example, in some hands mean F0 correlated positively with rated trustworthiness [20] and in other hands it was irrelevant [21,22]. Moreover, the direction of the F0 effect was shown to depend on context or the framing of the judgement task [23–26]. For example, in economic or mating-related contexts, voices with a higher F0 seemed more trustworthy than voices with a lower F0 irrespective of speaker sex. However, when probed more generally, impressions of trustworthiness appeared to differ by sex with low and high F0 being preferred for female and male voices, respectively [24].

Although past investigations of voice acoustics offered insights into the mechanisms underpinning vocal trustworthiness, more research is needed on how acoustically derived information about the speaker is relevant. Specifically, it is of interest to determine the extent to which expressive and person characteristics, and thus potentially higher-order conceptual processes, shape trustworthiness impressions. Evidence that they might comes from the face processing literature. Research on dynamic face characteristics indicates that positive or happy expressions are rated as more trustworthy than neutral expressions, which are perceived as more trustworthy than negative expressions such as anger [27,28]. The role of stable face characteristics has been explored by manipulating person characteristics such as sex and age. Although women consistently seem more trustworthy than men [29,30], results for age are mixed. In the context of a trust game, participants expressed greater trust towards older as compared with younger individuals [31], yet their trustworthiness ratings did not map onto actual behavior in the game. Moreover, other work assessing trustworthiness impressions found them to be independent of age [32] or to favour younger over older adults [33].

Effects of both speaker expressive and person characteristics on perceived trustworthiness have been explained by the “emotion over-generalization” hypothesis, first conceived as metaphorical generalization by Secord [34,35]. According to this framework, emotional expressions are important for signalling probable behaviors and because positive affect is believed to increase the likelihood of a benevolent intent it purportedly increases trust. Additionally, the similarity between emotional expressions and person characteristics is thought to explain why the latter are co-opted when judging whom to trust [36–38]. For example, average female faces have thinner inner eyebrows and rounder jaws than average male faces and this, among other features, boosts perceptions of positive affect or surprise and therefore, so the hypothesis suggests, of trustworthiness. Support for this, albeit not entirely consistent [39], comes from a number of studies on faces [38,40].

To date, only a couple of studies considered expressive and person characteristics in the context of vocal first impressions [10,41,42] and none, that we could identify, examined trustworthiness. Hence, we sought to address this issue and have done so in a previous publication of this data [43] that has since been retracted [44]. This original work was compromised by the fact that acoustic analyses of frequency and intensity related parameters were based on an entire utterance failing to exclude unvoiced elements such as voiceless consonants from processing. Therefore, the resulting values for statistical analysis were noisy. For this current manuscript we have revised the relevant acoustic analyses and optimized our statistical modelling. This corroborated the major results of our previous publication but changed some peripheral conclusions.

The study reported then and here employed 20 speakers, differing in age and sex, to produce two sentences in accordance with specific expressive instructions (see below). Each expression was subjected to an analysis of frequency, intensity and duration properties and to a trustworthiness rating by a group of 80 listeners. Statistical analyses tested the following hypotheses. (1) Expression valence was expected to predict perceived trustworthiness. As shown for faces, positive voices should be rated as more trustworthy than negative voices. We also pursued a possible, and not previously delineated, relation between the arousal and trustworthiness of vocal expressions. Besides valence, arousal, or a person’s level of energy, forms a major dimension in a basic affective space [45,46] and serves as a relevant cue to behavioral intentions. It may, therefore, also play a role in biasing trust. (2) A second hypothesis was that basic person characteristics like sex and age would modulate perceived trustworthiness. This was based on previous work showing that person characteristics bias trust preferences for faces [29,31]. In line with extant findings, we expected female voices to receive higher trustworthiness ratings than male voices. However, due to inconsistent results no directional predictions could be made for age effects. (3) Last, and in line with the emotion over-generalization hypothesis, we expected that acoustical parameters predicting higher perceived trustworthiness should also be the voice acoustics characterizing the expressive and person characteristics that are most trusted. For example, if a low mean F0 is associated with high perceived trustworthiness, then the affective expression, sex, and age receiving the highest trustworthiness ratings should have a lower mean F0 than the affective expression, sex, and age receiving the lowest trustworthiness ratings.

## Materials and methods

This research was approved by the Institutional Review Board of the National University of Singapore and conducted following established guidelines. Participants were informed about the study procedure and signed a consent form before commencing their participation.

### Participants

This study was conducted at the National University of Singapore and approved by its ethics review board. Singapore has a dominantly Chinese population and uses English as its official language. The sample size for this study was based on prior research exploring individual differences in voice perception [6,47]. Participants of this study were Singaporean residents who spoke English as their native language. Younger participants comprised 20 women with a mean age of 21.1 years (SD 2.05, 19–27) and 20 men with a mean age of 23.7 years (SD 3.3, 20–32). Older participants comprised 45 individuals of which five were excluded from data analysis because they could not follow task instructions (N = 2), because they could not hear the materials even after adjusting the volume (N = 1), or because they failed to complete the full session (N = 2). This left 20 older women with a mean age of 68 years (SD 5, 60–77) and 20 older men with mean age of 67.9 years (SD 7.1, 60–91). An ANOVA with the participants’ age as the dependent variable and Sex and Age Group as the independent variables revealed the expected Age Group main effect (F[1,75] = 1768.45, p < .0001) with the Sex effect (p = .27) and the interaction between Sex and Age Group (p = .209) being non-significant. Note that one young participant had to be excluded from this analysis because she failed to report her exact age.

Older participants completed the Mini Mental State Examination (MMSE) [48] and a hearing threshold test, which measured age-related decline in cognitive functioning and hearing, respectively. Older women scored an average of 29.2 points (SD 1.6) on the MMSE (normal range 24–30) and had a mean hearing threshold of 32.8 dB (SD 7.5). Older men scored an average of 29.4 (SD 0.9) on the MMSE and had a mean hearing threshold of 38.5 dB (SD 10). Hearing thresholds were determined by calculating mean hearing thresholds across both ears, and across all frequencies measured, i.e. 1000 Hz, 2000 Hz, 4000 Hz, 750 Hz, 500 Hz, 250 Hz, and 125 Hz. According to WHO standards (http://goo.gl/buEL92), two elderly participants had normal hearing (≤ 25dB), 28 had a slight hearing impairment (26 to 40dB), 9 had a moderate impairment (41 to 60dB) and one had a severe impairment (61 to 80dB) that was corrected with a hearing aid. To compensate for possible hearing difficulties, we asked participants to adjust the volume of sound presentations to a comfortable level.

### Stimuli

The stimuli used in this study were recorded from 20 Singaporean native English speakers with acting experience and are available on the Open Science Framework (https://osf.io/j3hfg/?view_only=77db09592fe44a79837f61ba8146fdb8). Speakers included five young women with a mean age of 22.2 years (range 21–24), five young men with a mean age of 23.8 years (range 23–25), five older women with a mean age of 69.2 years (range 62–85), and five older men with a mean age of 63 years (range 45–79). The older individuals were recruited from a local amateur-acting association, whereas the younger individuals were recruited through campus advertising. Acting experience ranged from 1 to 30 years (mean 5.75, SD 3.8) for the older speakers and from 1 to 6 years (mean 2.5, SD 1.65) for the younger speakers.

The number of speakers involved in this study was constrained by our ability to recruit mobile older individuals with acting experience. Although this number compares reasonably well with other work (please see Discussion for more details), it unfortunately limited our power in observing speaker effects.

Speakers attended individual recording sessions. They were given two English sentences (“The politician faced his audience.”, “The message reached its target.”) that the authors of this study deemed to be affectively neutral but that, depending on the context, could be potentially relevant for a range of affective experiences. Speakers were asked to produce these sentences with a range of expressive intentions. These expressive intentions were introduced to generate utterances with varying intonations and were deemed potentially relevant for modulating perceived trustworthiness by the authors of this manuscript. The specific voice conditions included six emotional expressions (i.e., content, happy, proud, afraid, angry, and sad) and four conversational expressions (i.e., confident, stating, doubtful, and questioning). Additionally, we asked speakers to attempt to sound trustworthy, untrustworthy, and neutral. For each of the 13 expressive intentions, speakers were given an expression label along with a short sentence describing a scenario for them to interpret vocally (e.g., trustworthy—You are talking to a stranger whom you wish to trust in you.). Speakers were asked to warm-up their voice by reading the sentences aloud neutrally. Subsequently, they could tackle the individual expressive intentions in their preferred order and repeat attempts until they were satisfied with their portrayal. Once they were satisfied with their portrayal, recordings were made with a few repetitions from which we later picked the clearest exemplar for each speaker, expressive intention, and sentence (e.g., no microphone artefacts, clear pronunciation, identifiable expressive intention). Recordings were conducted in a sound attenuated chamber and digitized at a 16 bit/44.1 kHz sampling rate. They were processed off-line using Adobe Audition 2.0. Specifically, individual sentences were cut into separate files and their intensity was normalized at the root-mean-square value.

**Fig 1:**
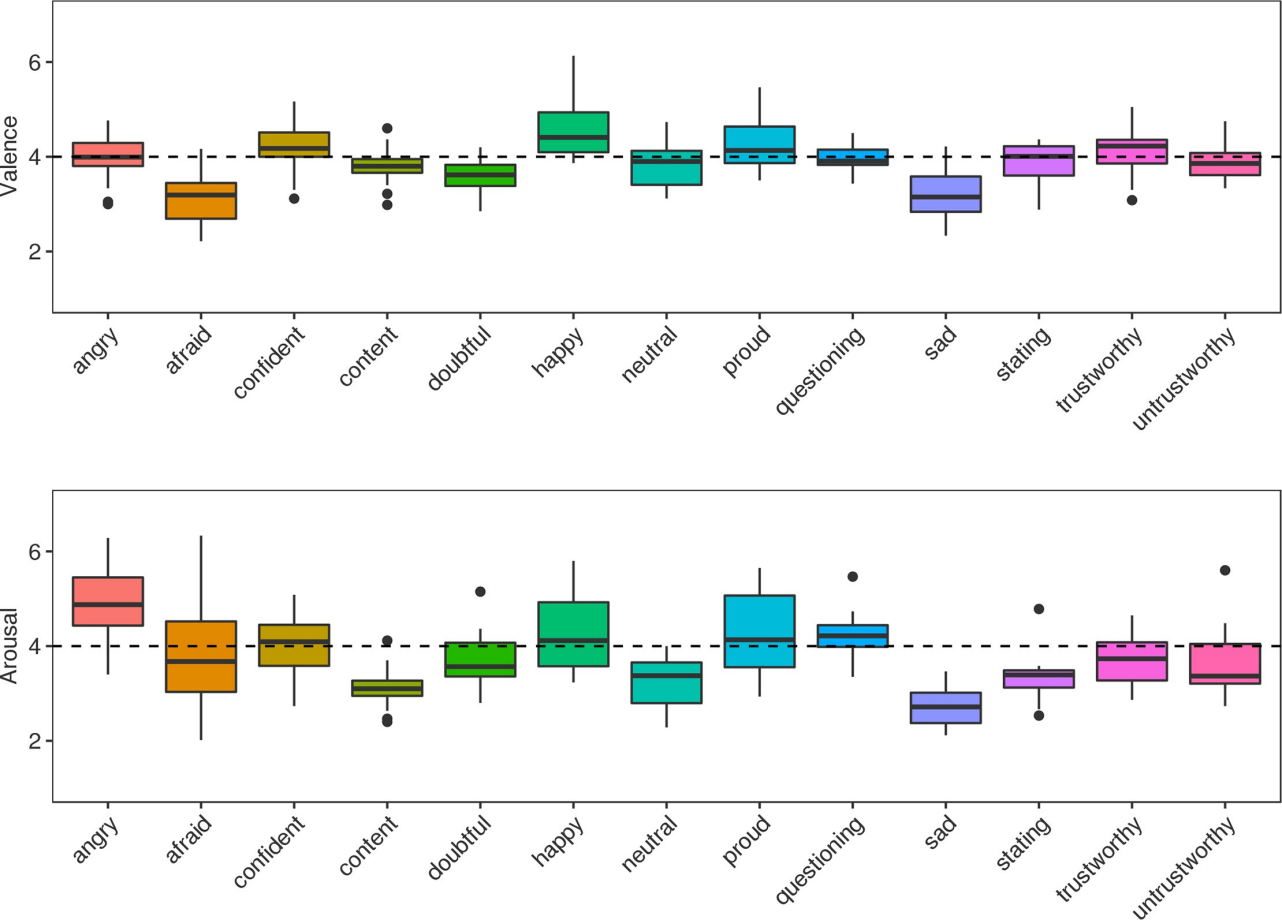
Box and whisker plots illustrating perceived valence (top) and perceived arousal (bottom) for each expression for a by-item analysis that used data points for each speaker and expression averaged across listeners and sentences. Black dashed lines indicate the rating scale midpoint.

In a preliminary rating study, 30 individuals (15 women, average age of 22 years (SD 3.77)) listened to each expression (2 sentences x 13 expressions x 20 speakers = 520) and rated its perceived valence and arousal on 7-point scales ranging from very negative to very positive and from very calm to very aroused. Valence and arousal ratings were subjected to separate ANOVAs with Expression (13 levels) as a repeated measures factor. This produced a significant main effect of Expression for both the Valence (F[12,348] = 44.25, p < .0001, gη^2^ = 0.481) and Arousal (F[12,348] = 74.99, p < .0001, gη^2^ = 0.466), indicating that there was considerable variation in how positive/negative and calm/excited the stimuli sounded. Rating results are illustrated in Fig 1.

Using Praat [49], we analysed the acoustic stimulus properties. Specifically, we measured eight parameters including sound duration (i.e., duration of the entire utterance), which may be considered a measure of speech rate as the number of syllables was kept constant across speakers and expressions (10 and 7 for sentences 1 and 2, respectively). The other parameters comprised harmonic-to-noise ratio (HNR; the periodicity of the signal; Harmonicity settings = 0.01/75/0.1/4.5, Time Step = 0.01 s, Minimum pitch = 75 Hz, Silence threshold = 0.1, Number of periods per window = 4.5), mean F0 (the lowest frequency band of an utterance; Time step: standard value 0.0, Pitch floor: standard value 75 Hz, Pitch ceiling: standard value 600 Hz), F0 range (the F0 difference between the lowest and the highest value in an utterance) and F0 standard deviation, intensity range (Intensity settings = 75/0/yes, Minimum Pitch = 75 Hz, Time Step = 0), jitter (i.e., short-term variability/perturbations in F0; Voice report settings = 0/0/75/600/1.3/1.6/0.03/0.45), and shimmer (i.e., short-term variability/perturbations in voice intensity; Voice report settings = 0/0/75/600/1.3/1.6/0.03/0.45). For all pitch and intensity related measures, we created grids marking the voiced sections but not the unvoiced sections for analysis.

Measurement values of all parameters were normalized (mean 0, SD 1) and subjected to a series of cumulative link mixed (CLM) models (i.e., ordered logit regression models) using the clmm function from the ordinal package [50] in R [51] with its default settings including the logit link function for data modelling and the Wald test for significance testing. In a first set of models, valence was the dependent variable, a single acoustic parameter was modelled as the fixed effect and the parameter slope and intercept for Raters and Speakers were modelled as random effects.

For valence, we observed, in order of effect size, a negative relationship with Duration (β = -.61, SE = .073, *Z* = -8.37, *p* < .0001) and HNR (β = -.6, SE = .08, *Z* = -7.75, *p* < .0001) and a positive relationship with mean F0 (β = .53, SE = .16, *Z* = 3.29, *p* < .001), F0 SD (β = .51, SE = .1, *Z* = 5.28, *p* < .0001), F0 range (β = .39, SE = .07, *Z* = 5.35, *p* < .0001), and Intensity Range (β = .13, SE = .04, *Z* = 3.12, *p* < .01). Effects for Jitter (β = .08, SE = .1, *Z* = .8, *p* = .43) and Shimmer were non-significant (β = .06, SE = .11, *Z* = .53, *p* = .59).

Analysing arousal in an analogous way revealed a negative relationship with HNR (β = -.83, SE = .11, *Z* = -7.64, *p* < .0001), Duration (β = -.48, SE = .08, *Z* = -5.98, *p* < .0001), and Jitter (β = -.43, SE = .09, *Z* = -4.96, *p* < .0001) and a positive relationship with mean F0 (β = 1.65, SE = .13, *Z* = 12.21, *p* < .0001), F0 SD (β = .86, SE = .09, *Z* = 9.41, *p* < .0001), F0 range (β = .75, SE = .08, *Z* = 9.15, *p* < .0001) and Intensity range (β = .3, SE = .09, *Z* = 3.17, *p* = .001). The Shimmer effect was non-significant (β = -.04, SE = .08, *Z* = -.57, *p* = .57).

Last we explored whether and how the two sex and age groups differed on the six acoustic parameters explored here. To this end, we fitted, for each between speaker variable, six linear mixed effect models using the lme4 package [52] in R [51] (in consideration of group size, the interaction between Speaker Age and Speaker Sex was not pursued). An acoustic parameter served as the dependent variable, Speaker Age or Speaker Sex served as fixed main effects, and the intercepts of Speaker and Expression served as random effects. Due to a limited number of data points, a more complex model including slopes in the Expression random effects term failed to consistently converge.

Compared to older voices, younger voices had, in order of effect size, a shorter utterance Duration (β = .46, SE = .08, *t* = 5.55, *p* < .0001) and a lower HNR (β = .42, SE = .13, *t* = 3.3, *p* = .004). Effects were non-significant for F0 SD (β = -.13, SE = .15, *t* = .85, *p* = .41), the Intensity range (β = -.002, SE = .13, *t* = -.01, *p* = .99), mean F0 (β = .07, SE = .19, *t* = .35, *p* = .73), F0 range (β = .13, SE = .16, *t* = .86, *p* = .4), Jitter (β = .07, SE = .16, *t* = .44, *p* = .67), and Shimmer (β = .11, SE = .16, *t* = .7, *p* = .5).

Compared to female voices, male voices had a lower mean F0 (β = .77, SE = .07, *t* = 10.85, *p* < .0001), a smaller F0 range (β = .64, SE = .06, *t* = 10.73, *p* < .0001), a lower F0 SD (β = .6, SE = .06, *t* = 10.07, *p* < .0001), more Shimmer (β = -.36, SE = .14, *t* = -2.56, *p* = .02) and more Jitter (β = -.32, SE = .15, *t* = -2.16, *p* = .04). All other effects were non-significant (Duration, β = .03, SE = .14, *t* = .26, *p* = .8; HNR, β = .2, SE = .15, *t* = 1.34, *p* = .2; Intensity range, β = .03, SE = .13, *t* = .19, *p* = .85).

### Procedure

After giving informed consent, participants provided their demographic information and received instructions from the experimenter. Specifically, they were informed that they would hear a series of utterances (2 sentences x 13 expressions x 20 speakers = 520) over headphones and should judge how trustworthy or untrustworthy the speaker sounded using a 7-point scale ranging from 1 (very untrustworthy) to 7 (very trustworthy). This rating scale was shown on screen following each sentence until participants selected a number and submitted their selection by pressing the enter key on a standard keyboard. The next trial started after 0.5 seconds. Each participant heard all utterances in random order.

### Statistical analysis

As was done for our stimulus analysis described above, the effects of our independent variables on perceived trustworthiness were explored using cumulative link mixed (CLM) models (i.e., ordered logit regression models) as implemented via the clmm function from the ordinal package [50] in R [51]. We used the function’s default settings including the logit link function for data modelling and the Wald test for significance testing. Analyses focused on our three questions of interest including the relation between trustworthiness and (i) speaker emotion (valence/arousal, (ii) speaker identity characteristics including age and sex, and (iii) acoustic features such a F0. For each question, we conducted CLM modelling with the relevant independent variable as the fixed effect and included its slope and intercept in the random effects term. For our non-categorical independent variables, we used scores normalized to a mean of 0 and a standard deviation of 1. Such an approach facilitates the interpretation of effect sizes provided by the β estimate. In the discussion section, we provide a more detailed review of effect sizes in the context of CLM modelling.

## Results

### Listener agreement

The data are available on the Open Science Framework (https://osf.io/j3hfg/?view_only=77db09592fe44a79837f61ba8146fdb8). We computed intraclass correlation coefficients (ICC) to determine listener agreement. To this end, we used the icc function from the irr package [53] in R [51] to examine two-way consistency across raters. This revealed that there was high agreement among participants in how they evaluated the voices (ICC(C,80) = .967).

### Trustworthiness and speaker affective expression

First, we examined the relationship between perceived trustworthiness—our ordinal dependent variable—and the affective rating of each item averaged across all raters (see Methods). Specifically, we fitted a CLM model (i.e., ordered logit regression model) with Trustworthiness as the dependent variable, either normalized Valence or Arousal (normed item values, mean 0, SD 1) as the fixed effect and the slopes and intercepts of Listener (40 levels) and Speaker (20 levels) as random effects. This analysis was conducted for young listeners only because only young listeners participated in the preliminary rating study from which we derived affective item norms.

**Fig 2:**
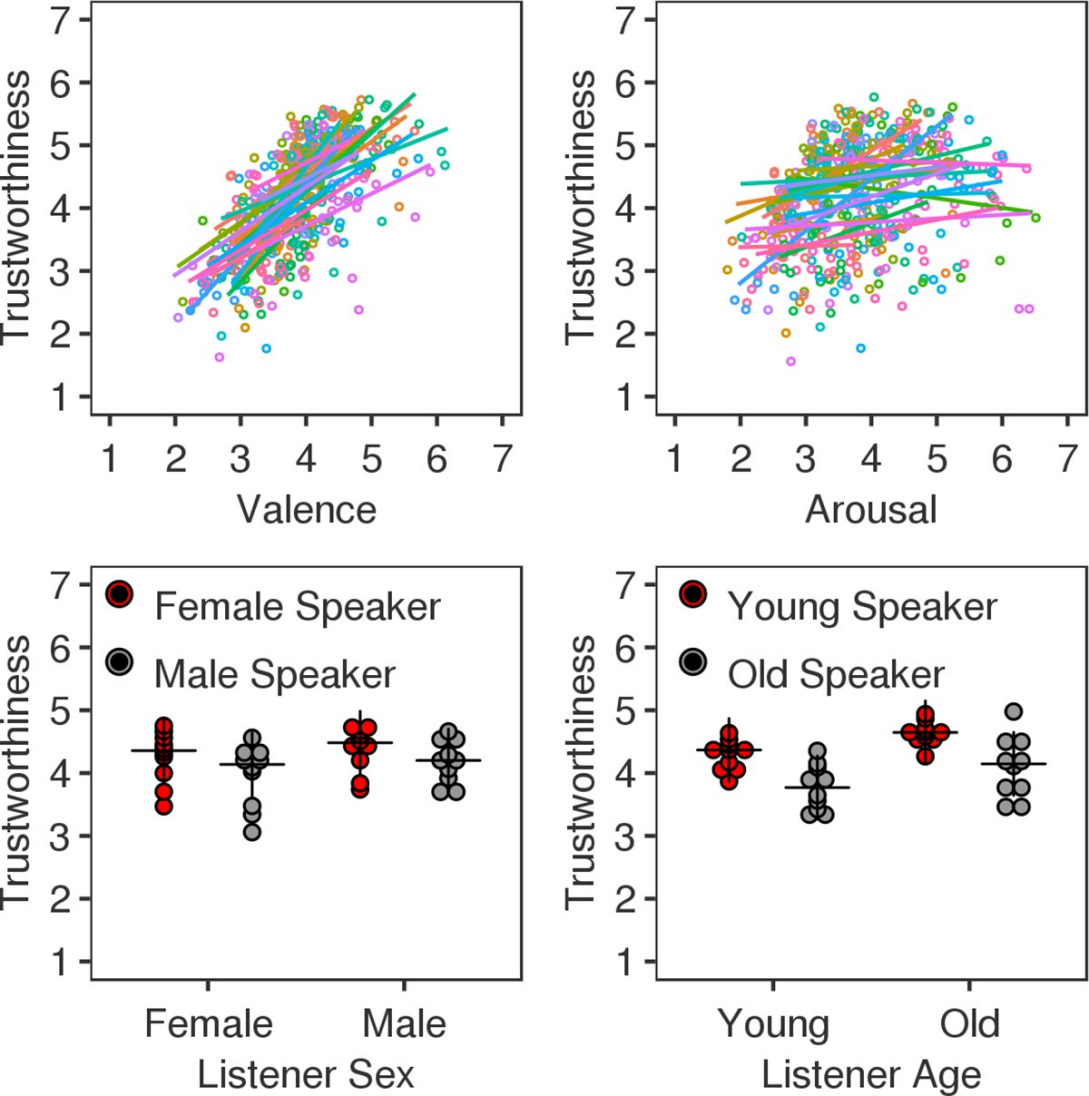
Trustworthiness rating results. Top row—The scatter plots illustrate the relation between valence and trustworthiness (left) and arousal and trustworthiness (right). Colours represent individual speakers (N = 20) in a consistent manner across the two graphs. Data points reflect trustworthiness averages for each expression and sentence computed across listeners and are summarized with a regression line. Bottom row–Plots illustrating the interaction of speaker and listener characteristics for sex (left) and age (right), respectively. Female and younger speakers are represented by red dots, whereas male and older speakers are represented by grey dots. Data points reflect trustworthiness averages for individual speakers computed across expressions, sentences and listeners. Median values are marked by a black cross.

As illustrated in Fig 2 (top row), perceived trustworthiness was positively related to Valence (β = .65, SE = .08, *Z* = 8.17, *p* < .0001) but unrelated to Arousal (β = .02, SE = .08, *Z* = .27, *p* = .79). Control analyses indicated that this pattern replicated well across the two sentences for Valence (sentence 1: β = .63, SE = .09, *Z* = 6.85, *p* < .0001; sentence 2: β = .75, SE = .11, *Z* = 6.84, *p* < .0001) but was less consistent for arousal (sentence 1: β = .16, SE = .07, *Z* = 2.15, *p* = .032; sentence 2: β = -.14, SE = .11, *Z* = -1.23, *p* = .218).

### Trustworthiness and person characteristics

The relationship between perceived trustworthiness and the person characteristics of speakers and listeners is illustrated in Fig 2 (bottom row). Trustworthiness ratings were again analysed using a CLM model with Listener Age (old/young), Listener Sex (female/male), Speaker Age (old/young) and Speaker Sex (female/male) as fixed effects. We modelled all main effects as well as the interactions between Listener Age and Speaker Age and between Listener Sex and Speaker Sex. We refrained from modelling other interactions because they were not relevant for our hypotheses and because we wished to keep the speaker number contributing to each effect at an acceptable level (N≥10). Fixed effects were complemented by a full random effects structure. The slopes of the speaker effects (Speaker Age, Speaker Sex) in combination with the intercepts of Listener (80 levels) as well as the slopes of the listener effects (Listener Age, Listener Sex) in combination with the intercepts of Speaker (20 levels) were random effects.

As expected, there were significant effects of Speaker Age (β = .6, SE = .16, *Z* = 3.71, *p* = .0002) and Speaker Sex (β = -.34, SE = .17, *Z* = -1.97, p = .05) pointing to a role of these variables in modulating listener trust. Trustworthiness ratings were higher for younger than for older voices and for female than for male voices. Additionally, there was a marginal Listener Age effect indicating that older listeners tended to give higher trustworthiness ratings than younger listeners (β = .39, SE = .23, *Z* = 1.66, *p* = .097). The Listener Sex effect was non-significant (β = .17, SE = .22, *Z* = .78, *p* = .44). The interactions involving Listener Sex and Speaker Sex (β = .08, SE = .08, *Z* = 1.01, p = .311) and Listener Age and Speaker Age (β = .05, SE = .18, Z = .27, *p* = .79) were also non-significant.

Control analyses conducted for each of the two sentences separately replicated the Speaker Age effect (sentence 1: β = .63, SE = .17, *Z* = 3.64, *p* = .0003; sentence 2: β = .54, SE = .19, *Z* = 2.91, *p* = .003) and, but less clearly, the Speaker Sex effect (sentence 1: β = -.41, SE = .17, *Z* = -2.44, *p* = .015; sentence 2: β = -.29, SE = .21, *Z* = -1.37, *p* = .169). Results, furthermore, suggested that the Listener Age effect was driven by sentence 2 (sentence 1: β = .21, SE = .25, *Z* = .84, *p* = .4; sentence 2: β = .59, SE = .24, *Z* = 2.44, *p* = .015).

### Acoustic features of trustworthiness

**Fig 3:**
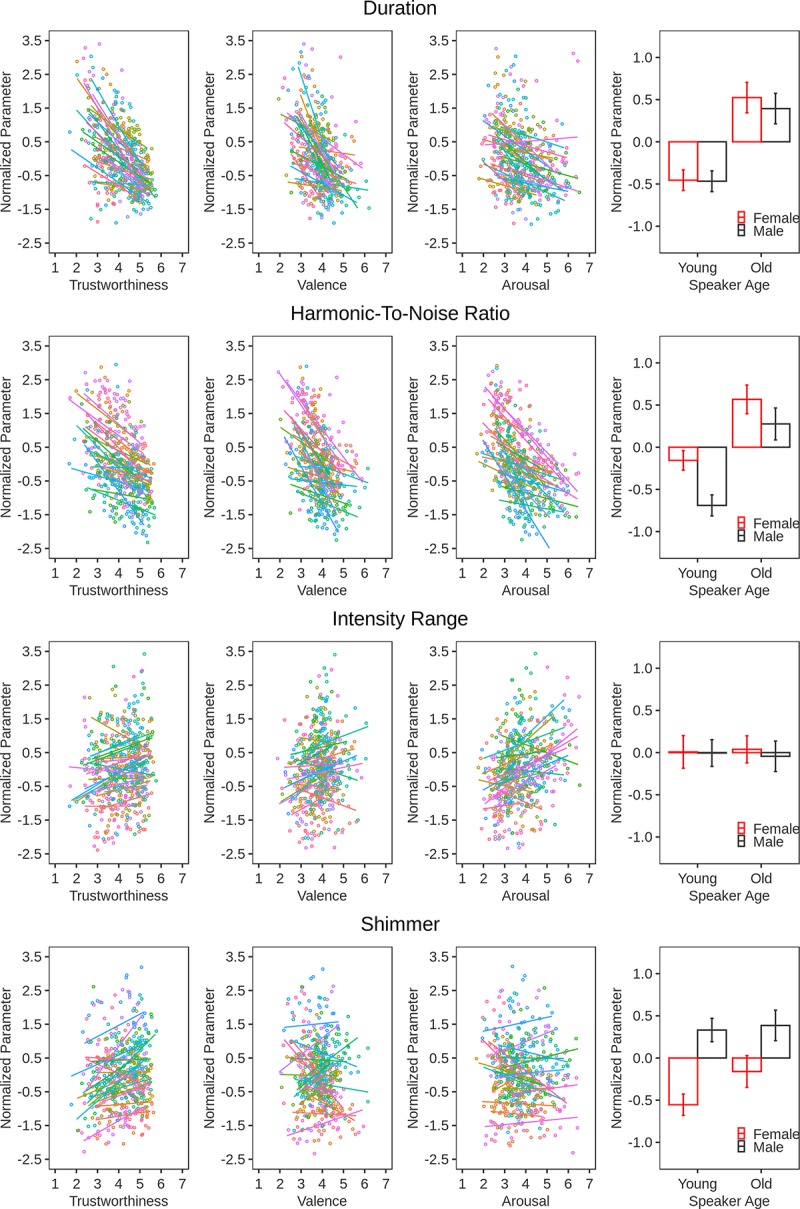
Illustration of the relationship between acoustic measures (i.e., utterance duration, HNR, intensity range, and shimmer) and other stimulus properties. Normalized vocal parameters (y-axes) are plotted as a function of trustworthiness ratings, valence, arousal, and speaker characteristics (x-axes). The individual points in the scatter plots represent the 13 expressions for the 2 sentences produced by each speaker. These points are summarized by a regression line for each speaker. The error bars in the bar-graphs represent the 95% confidence intervals.

**Fig 4:**
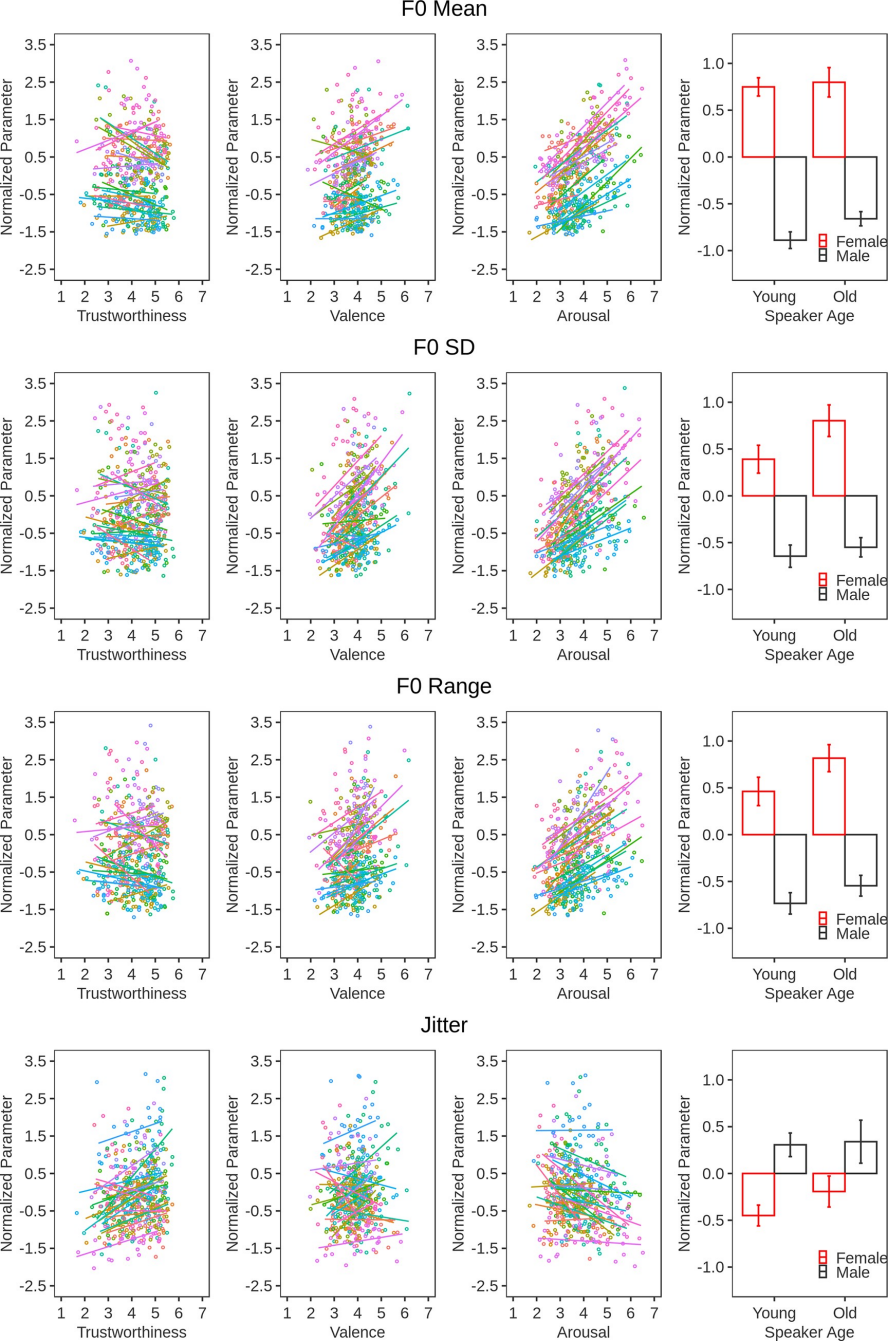
Illustration of the relationship between acoustic measures (i.e., F0 mean, F0 SD, F0 range, jitter) and other stimulus properties. Normalized vocal parameters (y-axes) are plotted as a function of trustworthiness ratings, valence, arousal, and speaker characteristics (x-axes). The individual points in the scatter plots represent the 13 expressions for the 2 sentences produced by each speaker. These points are summarized by a regression line for each speaker. The error bars in the bar-graphs represent the 95% confidence intervals.

We entered the normalized acoustic parameters into separate CLM models as the fixed main effect. Trustworthiness ratings served as the dependent variable and the slopes and intercepts of Listener and Speaker were random effects. The results are illustrated in Fig 3 and Fig 4 along with the stimulus properties detailed in the Methods section.

In order of effect size, higher trustworthiness was associated with a shorter utterance Duration (β = -.73, SE = .07, *Z* = -10.11, *p* < .0001), a lower HNR (β = -.55, SE = .09, *Z* = -5.87, *p* < .0001), more Shimmer (β = .37, SE = .08, *Z* = 4.40, p < .0001), a lower mean F0 (β = -.27, SE = .09, *Z* = -2.99, *p* = .003), more Jitter (β = .24, SE = .02, *Z* = 11.69 , p < .0001), and a larger Intensity range (β = .15, SE = .05, *Z* = 3.22 *p* = .001). Effects were non-significant for F0 range (β = -.05, SE = .06, *Z* = -.83, *p* = .4) and F0 SD (β = -.02, SE = .05, *Z* = -.3, *p* = .77).

We again conducted separate analyses for each sentence. This replicated the above effects for the two sentences for all measures (Duration S1, β = -.84, SE = .09, *Z* = -9.13, *p* < .0001; Duration S2, β = -.72, SE = .111, *Z* = -6.51, *p* < .0001; HNR S1, β = -.76, SE = .13, *Z* = -5.63, *p* < .0001; HNR S2, β = -.53, SE = .09, *Z* = -6.17, *p* < .0001; Shimmer S1, β = .47, SE = .1, *Z* = 4.58, *p* < .0001; Shimmer S2, β = .23, SE = .1, *Z* = 2.42, *p* = .015; Intensity Range S1, β = .23, SE = .08, *Z* = 2.8, *p* = .005; Intensity Range S2, β = .14, SE = .08, *Z* = 1.82, *p* = .069; Jitter S1, β = .36, SE = .17, *Z* = 2.18, *p* = .029; Jitter S2, β = .45, SE = .15, *Z* = 3.06, *p* = .002; F0 range S1, β = .05, SE = .1, *Z* = .46, *p* = .65; F0 range S2, β = -.13, SE = .08, *Z* = -1.63, *p* = .103; F0 SD S1, β = -.08, SE = .1, *Z* = .88, *p* = .377; F0 SD S2, β = -.09, SE = .07, *Z* = -1.27, *p* = .204) with the exception of mean F0 (S1: β = -.12, SE = .12, *Z* = -1, *p* = .31; S2, β = -.36, SE = .12, *Z* = -3.01, *p* = .003).

## Discussion

This study examined whether and in what way a speaker’s expressive and person characteristics shape listener trust.

Looking at expressive characteristics, we found a positive relationship between the valence of vocal expressions and rated trustworthiness. More positive voices were associated with increased perceived trustworthiness across our two stimulus sentences and across all speakers for the range of expressions that each speaker produced (Fig 2). Thus, we successfully replicate a phenomenon previously reported for faces [27,28]. Moreover, examination of our observed effect size points to a likely relevance of this phenomenon in everyday life. In the context of ordinal mixed effect modelling, we can expotentiate the regression coefficient β resulting in an odds ratio which indicates the factor by which the odds of obtaining a given dependent measure outcome would change with a unit increase/decrease in the independent measure. For the relation between valence and trustworthiness, we found that the predicted odds of observing a given trustworthiness rating j (j = 1 . 7) or higher when increasing utterance valence by 1 SD would increase by a factor of 1.91 (β = .65). Or expressed differently, the estimated probability of listeners perceiving an expression as more trustworthy increases by about 0.61 (exp(0.65/sqrt(2))/(1+exp(0.65/sqrt(2))), [54]) with an increase of 1 SD in perceived valence. Please note that for odds ratios larger than 1, a larger value signifies a larger positive effect, whereas for odds ratios smaller than 1, a smaller value signifies a larger negative effect.

Notably, the arousal of vocal expressions failed to predict perceived trustworthiness (β = .02). There was a small positive relation for one (“The politician faced his audience.”) and a non-significant relation for the other (“The message reached its target.”) sentence suggesting some dependence on verbal context and potential subtle differences in affective biases between the sentences. Moreover, mean F0 was, of all acoustic parameters, most strongly associated with arousal and this association was by far the largest one in this study (β = 1.69, exp(β) = 5.21). However, mean F0 predicted trustworthiness less strongly and in the opposite direction (β = -.27, exp(β) = .76). Together the mixed relation between rated arousal and trustworthiness and the differentiation in underlying voice acoustics suggests that the role of arousal in perceived trustworthiness may be negligible.

The present study examined the role of person characteristics for trust from voices by looking at speaker sex and age effects. Extending evidence from faces [29,30], vocal recordings of women were rated as more trustworthy than those of men. This pattern showed consistently across the two sentences used here (β = -.41, -.29) but was significant for only one of them–when speakers said “The politician faced his audience.” but not when they said “The message reached its target.”. Thus, although the female trustworthiness advantage may be a fairly robust phenomenon, its presence is moderated by other variables. For example, its strength may change as a function of whether trust concerns truthfulness or capability. In line with this, prevailing stereotypes characterize women as warmer but less competent than men [55].

Unlike our speaker sex effect, our speaker age effect was significant for both sentences. Additionally, it was larger (β = .59, exp(β) = 1.81) than the speaker sex effect (β = -.33, exp(β) = .71) suggesting a greater relevance of age than sex in everyday trust. The age effect direction was both in conflict and in agreement with past research, which has provided a mixed pattern so far. Specifically, it was in conflict with work by Bailey and colleagues who found that older adult faces elicited higher trustworthiness ratings than younger adult faces [31]. However, those ratings were obtained after participants engaged in a trust-game with older and younger players and may, hence, have been context specific. Moreover, the age of the stimulus faces was left unspecified and may have been closer to middle age than to old age. This possibility agrees with research on political voting showing a preference for candidates in their 40s and 50s over younger and older candidates [15].

The present results corroborate evidence that younger faces appear more trustworthy than older faces [33] and that perceived vocal babyishness positively predicts ratings of warmth, honesty, and kindness [41]. Additionally, they converge with a more general age bias and a “youth ideal” that appeals on multiple levels and that readily engages processing resources. For example, research has shown that compared to expressions from older adults, expressions from younger adults more readily capture attention [56] and evoke better nonverbal perception [6]. Additionally, in surveys, younger individuals are seen as having better interpersonal skills as well as greater potential for professional development [57].

By examining both speaker age and sex, the present study also explored a potential role of peer status for perceived trustworthiness. Previous research identified own-age and own-sex preferences. Interpersonal attitudes have been characterized as more positive towards own as compared to other age individuals [57,58]. Additionally, children develop an own-sex bias that weakens after adolescence, but nevertheless persists into adulthood [59]. Together, this evidence raises the possibility that own-age and own-sex individuals are perceived as more trustworthy than those of another age and sex.

In line with prior work on facial age [32], however, the present study rejects this possibility in the context of vocal age. Younger and older listeners showed a similar age bias suggesting that youth is more relevant than peer status when it comes to trust. Likewise, there was no indication for a role of peer effects in modulating sex-based preferences. Specifically, the preference for female over male voices was comparable between women and men. One possible explanation for these null results is that participants may have felt anonymous making their peer status irrelevant. Future research may probe this possibility with virtual or face-to-face interactions in which participants are perceived by the speaker.

The present speaker sex effects are relevant in the context of recent work presenting untrustworthy, neutral, and trustworthy female and male faces [60]. Compared to men, women rated trustworthy faces as more trustworthy especially when they were female. Because we pursued sex effects across voices irrespective of their trustworthiness, we speculated that a similar interaction between sender and receiver sex may emerge in a re-analysis of only trustworthy voices (rating > 4). This possibility, however, was not supported (p = .97). Thus, more research is needed to determine whether a sex-based peer effect for vocal trustworthiness may be identified with a larger number of speakers and listeners.

Both speaker expressive and person characteristics shaped voice acoustics and, through this, biased perceptions of trustworthiness. To see how, we measured voice acoustics and linked them to both the trustworthiness ratings and the speaker variables (i.e., expression, age, sex). In doing so, we followed previous work on emotion expression [4] and examined a range of parameters describing frequency, intensity, and temporal sound properties.

Supporting earlier studies [15–18], we found that mean F0 negatively predicted trustworthiness. In other words, utterances with a lower speech melody were perceived as more trustworthy than utterances with higher speech melody. Notably, however, this relationship has not been shown consistently [20–22] and was somewhat volatile in the present data. In the analysis reported here, the mean F0 effect was significant for one of the stimulus sentences only. Moreover, it emerged only after we carefully examined and cleaned Praat F0 traces of small F0 artefacts caused, most likely, by fry or percussive mouth actions speakers added to voicing. Thus, it is not a very robust effect and in size (β = -.27, exp(β) = .76) smaller than that of several other acoustic parameters including duration (β = -.73, exp(β) = .49) and HNR (β = -.55, exp(β) = .57), which also negatively predicted trustworthiness, as well as shimmer (β = .37, exp(β) = 1.44) and jitter (β = .24, exp(β) = 1.27), which had a positive effect.

Looking at the acoustics of speaker affective expression, we found some overlap with those of trustworthiness. Like trustworthy vocalizations, more positive vocalizations were associated with a shorter utterance duration and a lower HNR. However, contrary to the over-generalization hypothesis they were also characterized by a higher F0 mean, a larger F0 SD, a larger F0 range, a larger intensity range and no effects for jitter and shimmer. Additionally, person characteristics produced mixed results. Looking at age, we found that, as predicted by the overgeneralization hypothesis, younger individuals spoke faster and with a lower HNR than older individuals. However, other parameters had non-significant effects. Looking at sex, we found that, as predicted by the overgeneralization hypothesis, women expressed greater jitter and shimmer. However, in counter-evidence their voices also had a higher mean F0, a larger F0 SD, a larger F0 range and were comparable to those of men in duration, HNR, and intensity range.

Overall, the present study offers some support for the emotion over-generalization hypothesis [40,38], but see [39]. Specifically, it highlights speech rate and HNR as being relevant in the link between perceived trustworthiness and positive affect and suggests that the greater perceived trustworthiness of younger as compared to older individuals may be due to their speech rate and HNR positively biasing emotion perception. Yet, as detailed above many acoustical parameters contribute to perceptions of trustworthiness and most of them dissociated from perceptions of speaker affect, age and sex offering no support for the emotion overgeneralization hypothesis. Thus, positive affect is limited in explaining first impressions of trustworthiness. Instead, these impressions seem to emerge multi-causally. Some of their mechanisms may be largely perceptual and ‘hard-wired’ and tap on the biological convergence of expressive and person characteristics. However, others may depend on the more flexible, higher-order conceptual knowledge that individuals acquire across their life-time (e.g., stereotypes).

Although the present study provides insights into how vocal expressive and person characteristics shape trustworthiness impressions, it also raises questions for future research. One such question concerns the speaker sex and age effects observed here. The current speaker sample, albeit comparable to much published work [15–21,23–26], was small and one must worry that outliers biased overall effects and between speaker comparisons. We addressed this issue by controlling for individual speakers in our statistical analysis and by exploring whether effects emerge consistently for speakers across, as well as within, sex and age groups (Fig 2). As this was the case, we are confident in our results. Nevertheless, one may wish to replicate them with a larger and more representative sample.

A second question is how age affects both the vocal conveyance of trustworthiness and its perception. Looking at vocal production, it is still unclear how ageing changes the voice and the mechanisms of vocal expression. For example, although there is evidence that mean F0 increases in older adults [61] this evidence is inconsistent [62] and may interact with generational or cultural differences in life-style (e.g., smoking, singing) [62,63]. Additionally, it would be interesting to consider age differences in the motivation and ability to pose expressions and to examine spontaneous utterances as a function of age. Looking at perceiving trustworthiness, research should address the relevance of sensory, cognitive and emotional changes associated with ageing. For example future work may consider how age related hearing loss, decreasing executive functions [6], or a greater emphasis on positive experiences [64] may boost perceptions of trustworthiness in older adults.

Last, it would be important to pursue the role of voices for perceiving trustworthiness in everyday life. One issue is whether perceptions are misguiding or represent useful biases in situations with limited information. Clearly, we cannot be exceedingly accurate in judging trustworthiness from nonverbal cues only [1], but see [65]. Yet, there is empirical data suggesting that such judgements may not be entirely off the mark. Trustworthiness appears to have a stable personality basis [66] and observers appear to gauge some rudimentary aspects of this from faces more accurately than would be expected by chance [67–69]. Thus, one next step would be to explore the validity of trust impressions from the voice. Additionally, it would be relevant to examine how impressions inform actual behaviors.

## Conclusions

To conclude, the present study pursued voice-based trustworthiness impressions from a fresh angle. In addition to exploring the sound properties of trustworthiness, it examined the role of vocal expressive and person characteristics and linked those back to relevant voice acoustics. In doing so, it produced original evidence that positive voices sound more trustworthy than negative voices. Additionally, it delineated speaker age and sex effects according to which trustworthiness is higher for younger as compared with older and female as compared with male individuals. Last, this study highlighted speech rate and HNR as trust’s most important acoustic dimensions and showed that they map onto a subset of expressive and person characteristics providing some, albeit imperfect, support for the emotion overgeneralization hypothesis. Notably, a single acoustic parameter—fast speech—was more important than speaker affect, age, and sex in predicting listener trust thus underlining that trust likely arises from both simple perceptual as well as higher-order conceptual processes.
